# Influence of proton pump inhibitors on the incidence and clinical course of oesophageal fistula following catheter ablation for atrial fibrillation: a subanalysis of the POTTER-AF study

**DOI:** 10.1093/europace/euaf212

**Published:** 2025-10-24

**Authors:** Thomas Beiert, Vincent Knappe, Andreas Zietzer, Vanessa Schmidt, Helmut Pürerfellner, Philipp Sommer, Christian Sohns, Christian Veltmann, Daniel Steven, Kyoung-Ryul Julian Chun, Philippe Maury, Estelle Gandjbakhch, Mikael Laredo, Stephan Willems, Martin Borlich, Anna Füting, Raphael Spittler, Sergio Richter, Anja Schade, Malte Kuniss, Carsten Wunderlich, Dong-In Shin, Dirk Grosse Meininghaus, Marc Bonsels, David Reek, Uwe Wiegand, Alexander Bauer, Andreas Metzner, Lars Eckardt, Olaf Krahnefeld, Christian Sticherling, Michael Kühne, Dinh Quang Nguyen, Laurent Roten, Dominik Linz, Pepijn van der Voort, Bart A Mulder, Johan Vijgen, Alexandre Almorad, Charles Guenancia, Laurent Fauchier, Serge Boveda, Yves De Greef, Antoine Da Costa, Pierre Jais, Antoine Milhem, Laurence Jesel, Rodrigue Garcia, Hervé Poty, Ziad Khoueiry, Julien Seitz, Julien Laborderie, Alexis Mechulan, Francois Brigadeau, Alexandre Zhao, Yannick Saludas, Olivier Piot, Nikhil Ahluwalia, Claire A Martin, Jian Chen, Bor Antolic, Georgios Leventopoulos, Emin Evren Özcan, Hikmet Yorgun, Serkan Cay, Kivanc Yalin, Maichel Sobhy Botros, Ewa Jędrzejczyk-Patej, Osamu Inaba, Ken Okumura, Koichiro Ejima, Houman Khakpour, John N Catanzaro, Vivek Reddy, Andrea Natale, Hermann Blessberger, Bing Yang, Julia Vogler, Karl-Heinz Kuck, José Luis Merino, Ahmad Keelani, Christian-H Heeger, Sorin S Popescu, Roland Richard Tilz

**Affiliations:** Heart Center Bonn, Department of Internal Medicine II, University Hospital Bonn, Venusberg-Campus 1, Bonn D-53127, Germany; Heart Center Bonn, Department of Internal Medicine II, University Hospital Bonn, Venusberg-Campus 1, Bonn D-53127, Germany; Heart Center Bonn, Department of Internal Medicine II, University Hospital Bonn, Venusberg-Campus 1, Bonn D-53127, Germany; Department of Rhythmology, University Heart Center Lübeck, University Hospital Schleswig-Holstein, Ratzeburger Allee 160, Lübeck D-23538, Germany; Institute for Diagnostic and Interventional Radiology, Faculty of Medicine and University Hospital Cologne, University of Cologne, Cologne, Germany; Interne 2, Ordensklinikum Linz Elisabethinen, Linz, Austria; Kliniken für Elektrophysiologie/Rhythmologie, Herz- und Diabeteszentrum NRW, Universitätsklinik der Ruhr-Universität Bochum, Bad Oeynhausen, Germany; Kliniken für Elektrophysiologie/Rhythmologie, Herz- und Diabeteszentrum NRW, Universitätsklinik der Ruhr-Universität Bochum, Bad Oeynhausen, Germany; Heart Center Bremen, Electrophysiology Bremen, Bremen, Germany; Department for Electrophysiology, Heart Center University Cologne, Cologne, Germany; MVZ CCB am Agaplesion Markus Krankenhaus, Frankfurt a.M., Germany; Department of Cardiology, University Hospital Rangueil, Toulouse, France; Sorbonne Université, APHP, Pitié Salpêtrière University Hospital, Cardiology Institute, Paris, France; Sorbonne Université, APHP, Pitié Salpêtrière University Hospital, Cardiology Institute, Paris, France; Klinik für Kardiologie und Internistische Intensivmedizin, Asklepios Klinik St. Georg, Hamburg, Germany; Heart Center, Segeberger Kliniken (Academic Teaching Hospital of the Universities of Kiel, Lübeck and Hamburg), Bad Segeberg, Schleswig-Holstein, Germany; Department of Electrophysiology, Alfred Krupp Hospital, Essen, Germany; Department of Medicine, Witten/Herdecke University, Witten, Germany; Department of Cardiology II/Electrophysiology, Center for Cardiology, University Hospital Mainz, Mainz, Germany; Department of Internal and Cardiovascular Medicine, Herzzentrum Dresden, University Clinic, Technische Universität Dresden, Dresden, Germany; Department of Interventional Electrophysiology, Helios Hospital Erfurt, Erfurt, Germany; Department of Rhythmology, Rhoen Klinikum Campus Bad Neustadt/Saale, Bad Neustadt/Saale, Germany; Department of Cardiology, Kerckhoff Heart Center, Bad Nauheim, Germany; Helios Klinikum Pirna, Klinik für Innere Medizin II, Pirna, Germany; Department of Medicine, Witten/Herdecke University, Witten, Germany; Department of Cardiology, Heart Centre Niederrhein, Helios Clinic Krefeld, Krefeld, Germany; Department of Cardiology, Medical University Lausitz - Carl Thiem, Cottbus, Germany; Klinik für Kardiologie, Kliniken Maria Hilf GmbH, Mönchengladbach, Germany; Department of Cardiology, University Hospital Augsburg, Augsburg, Germany; Sana-Klinikum Remscheid GmbH, Akademisches Lehrkrankenhaus der Universität zu Köln, Remscheid, Germany; Innere Medizin I, Diak-Klinikum Schwäbisch Hall und Klinikum Crailsheim, Schwäbisch Hall, Germany; Klinik für Kardiologie, Universitäres Herz- und Gefäßzentrum, Universitätsklinikum Hamburg-Eppendorf, Hamburg, Germany; Department of Cardiology II (Electrophysiology), University Hospital Münster, Münster, Germany; Medizinische Klinik II, Sana Kliniken Lübeck, Lübeck, Germany; Deaprtment of Cardiology, University Hospital Basel, Basel, Switzerland; Deaprtment of Cardiology, University Hospital Basel, Basel, Switzerland; Kardiologie und Rhythmologie, St. Vinzenz-Hospital Köln, Köln, Germany; Department of Cardiology, Inselspital, Bern University Hospital, University of Bern, Bern, Switzerland; Department of Cardiology, Maastricht University Medical Center and Cardiovascular Research Institute Maastricht, Maastricht, The Netherlands; Cardiology, Catharina Hospital, Eindhoven, The Netherlands; Department of Cardiology, University of Groningen, University Medical Center Groningen, Groningen, The Netherlands; Heart Center Hasselt, Jessa Hospital, Hasselt, Belgium; Heart Rhythm Management Centre, Postgraduate Program in Cardiac Electrophysiology and Pacing, Universitair Ziekenhuis Brussel - Vrije Universiteit Brussel, European Reference Networks Guard-Heart, Brussels, Belgium; Cardiology Department, Dijon University Hospital, Dijon, France; Service de Cardiologie, Centre Hospitalier Universitaire Trousseau, Tours, France; Heart Rhythm Management Centre, Postgraduate Program in Cardiac Electrophysiology and Pacing, Universitair Ziekenhuis Brussel - Vrije Universiteit Brussel, European Reference Networks Guard-Heart, Brussels, Belgium; Cardiology – Heart Rhythm Management Department, Clinique Pasteur, Toulouse, France; Heart Rhythm Management Centre, Postgraduate Program in Cardiac Electrophysiology and Pacing, Universitair Ziekenhuis Brussel - Vrije Universiteit Brussel, European Reference Networks Guard-Heart, Brussels, Belgium; Department of Cardiology, ZNA Heart Centre, Antwerp, Belgium; Division of Cardiology, Jean Monnet University, Saint-Etienne, France; CHU Bordeaux, Univ. Bordeaux, IHU LIRYC ANR-10-IAHU-04, Bordeaux, France; Cardiology Department, La Rochelle Hospital, La Rochelle, France; Division of Cardiovascular Medicine, University Hospital Strasbourg, Srasbourg, France; Department of Cardiology, University Hospital of Poitiers, Poitiers, France; Centre d’Investigation Clinique 1402, University Hospital of Poitiers, Poitiers, France; Cardiologie, Clinique Tonkin, Lyon, France; Service de Cardiologie, Clinique Saint Pierre, Perpignan, France; Electrophysiology, Hospital St. Joseph, Marseille, France; Cardiology Department, Bayonne Hospital, Bayonne, France; Service de Cardiologie, Hospital Clairval, Marseille, France; Service Cardiologie, University Hospital Lille, Lille, France; Cardiologie et Vasculaire, Clinique Ambroise Parée, Paris, France; Cardiologie, Clinique Pôle Santé République, Clermont Ferrand, France; Centre Cardiologie du Nord, Saint Denis, France; Barts Heart Centre, Barts Health NHS Trust, London, UK; Wiliam Harvey Heart Centre, Queen Mary University of London, London, UK; Department of Cardiology, Royal Papworth Hospital, University of Cambridge, Cambridge, UK; Department of Heart Disease, Haukeland University Hospital, University of Bergen, Bergen, Norway; Department of Cardiology, University Medical Center Ljubljana, Ljubljana, Slovenia; Department of Cardiology, University of Patras, Patras, Greece; Heart Rhythm Management Center, Dokuz Eylul University, Izmir, Turkey; Department of Cardiology, Hacettepe University, Ankara, Turkey; Department of Cardiology, Division of Arrhythmia and Electrophysiology, University of Health Sciences, Yuksek Ihtisas Cardiovascular Building, Ankara City Hospital, Ankara, Turkey; Department of Cardiology, Cerrahpasa Faculty of Medicine, Istanbul University-Cerrahpasa, Istanbul, Turkey; Department of Critical Care Medicine, Faculty of Medicine, Cairo University, Cairo, Egypt; Department of Cardiology, Congenital Heart Diseases and Electrotherapy, Silesian Centre for Heart Diseases, Zabrze, Poland; Department of Cardiology, Japanese Red Cross Saitama Hospital, Saitama, Japan; Division of Cardiology, Saiseikai Kumamoto Hospital, Kumamoto, Japan; Department of Cardiology, Tokyo Women’s Medical University, Shinjuku-ku, Tokyo, Japan; UCLA Cardiac Arrhythmia Center, David Geffen School of Medicine at UCLA, Los Angeles, USA; Department of Cardiovascular Sciences, Brody School of Medicine, East Carolina University Health, Greenville, NC, USA; Helmsley Electrophysiology Center, Mount Sinai Fuster Heart Hospital, Icahn School of Medicine at Mount Sinai, New York, NY, USA; Texas Cardiac Arrhythmia Institute, St. David’s Medical Center, Austin, TX, USA; Department of Biomedicine and Prevention, Division of Cardiology, University of Tor Vergata, Rome, Italy; Metro Health Medical Center, Case Western Reserve University School of Medicine, Cleveland, OH, USA; Department of Cardiology, Kepler University Hospital, Linz, Austria; Department of Cardiology, Shanghai East Hospital, Tongji University, Shanghai, China; Department of Rhythmology, University Heart Center Lübeck, University Hospital Schleswig-Holstein, Ratzeburger Allee 160, Lübeck D-23538, Germany; Klinik für Kardiologie und Internistische Intensivmedizin, Asklepios Klinik St. Georg, Hamburg, Germany; Department of Rhythmology, University Heart Center Lübeck, University Hospital Schleswig-Holstein, Ratzeburger Allee 160, Lübeck D-23538, Germany; La Paz University Hospital, Universidad Autónoma de Madrid, Idipaz, Madrid, Spain; Department of Rhythmology, University Heart Center Lübeck, University Hospital Schleswig-Holstein, Ratzeburger Allee 160, Lübeck D-23538, Germany; Department of Rhythmology, University Heart Center Lübeck, University Hospital Schleswig-Holstein, Ratzeburger Allee 160, Lübeck D-23538, Germany; German Center for Cardiovascular Research (DZHK), Partner Site Hamburg/Kiel/Lübeck, Lübeck, Ratzeburger Allee 160, 23538 Lübeck, Germany; Department of Rhythmology, University Heart Center Lübeck, University Hospital Schleswig-Holstein, Ratzeburger Allee 160, Lübeck D-23538, Germany; German Center for Cardiovascular Research (DZHK), Partner Site Hamburg/Kiel/Lübeck, Lübeck, Ratzeburger Allee 160, 23538 Lübeck, Germany; Department of Rhythmology, University Heart Center Lübeck, University Hospital Schleswig-Holstein, Ratzeburger Allee 160, Lübeck D-23538, Germany; German Center for Cardiovascular Research (DZHK), Partner Site Hamburg/Kiel/Lübeck, Lübeck, Ratzeburger Allee 160, 23538 Lübeck, Germany

**Keywords:** Atrial fibrillation, Catheter ablation, Oesophageal fistula, Proton pump inhibitor

## Introduction

Atrial fibrillation (AF) represents the most common cardiac arrhythmia with an increasing incidence and prevalence worldwide.^1^ Pulmonary vein isolation (PVI) via catheter ablation is the cornerstone of AF treatment.^1^ Despite years of experience and significant technological advances, oesophageal fistula (OF) remains the most severe complication of catheter ablation for AF, associated with high morbidity and mortality.^2,3^ The POTTER-AF study reported a very low incidence of 0.025%.^2^ Therefore, data on effective preventive measures remain limited. Despite the lack of evidence, proton pump inhibitor (PPI) therapy is a widely adopted prophylactic treatment.^4,5^ While PPIs are considered to be generally well tolerated, recent studies have demonstrated relevant pharmacological interactions and adverse effects, warranting a cautious prescription.^6^ The aim of this study was to evaluate the impact of routine PPI use on the incidence and clinical course of OF in the POTTER-AF study.

## Methods

All patients diagnosed with an OF from the POTTER-AF study were stratified based on the use of post-procedural PPI.

Normally distributed variables are reported as mean ± standard deviation. Non-normally distributed variables are shown as median and interquartile range. The unpaired Student’s *t*-test was conducted for group comparisons if normally, and the Mann–Whitney U test if non-normally distributed. Categorical variables are displayed as absolute numbers and relative frequencies and were compared using Fisher’s exact test.

## Results

### Routine proton pump inhibitor prescription

**Figure 1:**
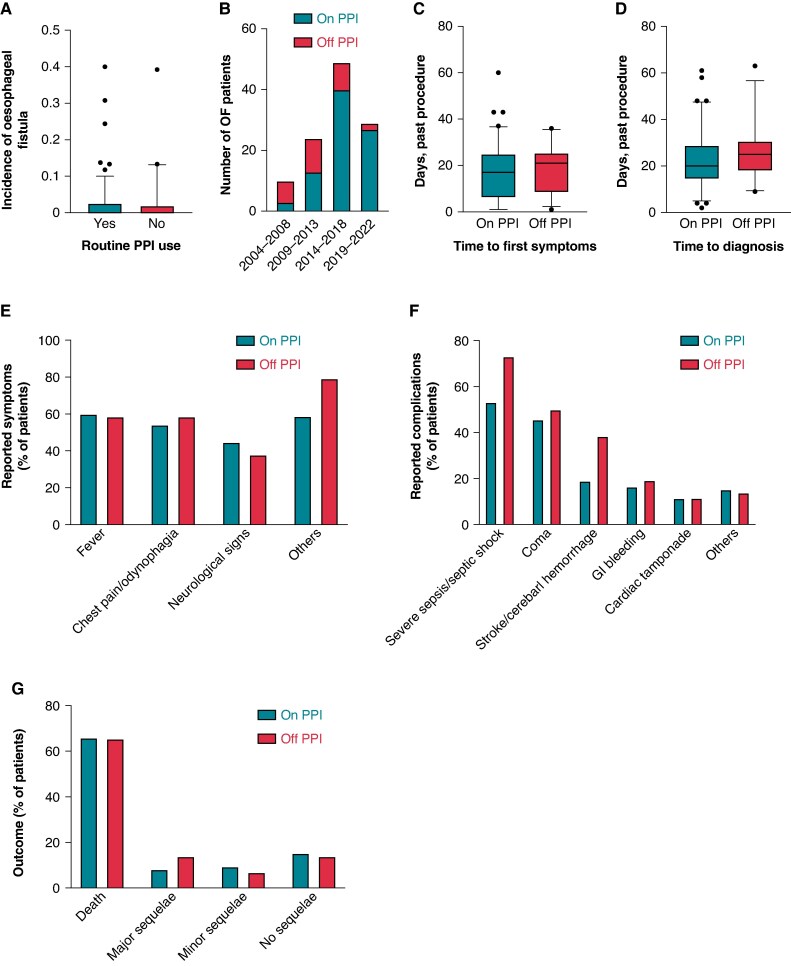
(*A*) Incidence of OF in relation to institutional routine post-procedural PPI treatment. Data points represent individual institutions. (*B*) Development of PPI prescription in OF patients over time. Displayed are numbers of OF patients with and without PPI prescription in indicated time frames. (*C* and *D*) Time to first symptoms (*C*) and time to diagnosis (*D*). Data points represent individual patients. (*E*) Overview of symptoms on first clinical presentation. (*F*) Overview of complications. (*G*) Overview of the outcome of all patients with OF. PPI, proton pump inhibitor; GI, gastrointestinal. The bottom and top edges of the box plots represent the 25th and 75th percentiles, and the lower and upper whiskers give the 5th and 95th percentiles, respectively. The lines within the boxes indicate the median values.

Of the participating centres, 195 of 214 had available data on institutional routine post-procedural PPI prescription. In 155 centres (79.5%), patients were routinely treated with PPI after an AF ablation procedure. The mean rate of OF in those centres was 0.023% ± 0.053%, compared to 0.024% ± 0.067% in centres without routine PPI treatment (*P* = 0.842; *Figure 1A*).

Throughout the study period, we observed an increase in PPI prescription in the cohort of OF patients, rising from 30% before 2009 to 93% after 2018 (*Figure 1B*).

### Patient population

Data on periprocedural characteristics, management, outcome and information on post-procedural PPI prescription were available for 114 patients with OF. Patients had a mean age of 62.5 ± 11.4 years. Paroxysmal, persistent, and long-standing persistent AF were present in 43%, 50%, and 7% of patients, respectively. Eighty-five patients (75%) were treated with PPI after the ablation procedure. The proportion of female patients was significantly lower in the cohort with PPI compared to the group without (41.2% vs. 67.9%, *P* = 0.017). No other differences were noted.

The energy source used was radiofrequency in 96.5%, cryoballoon in 2.6%, and laser balloon in 0.6% of patients, with no significant differences between the groups. Contact force measuring catheters were used more often in the ‘on PPI’ group (56.3% vs. 17.2%, *P* < 0.001). Additional linear ablations in the left atrium were performed in 45.7% of patients.

### Patient presentation

The median time between procedure and onset of symptoms was comparable between groups (17.0 (6.0, 25.0) days vs. 21.0 (8.3, 25.5) days for patients ‘on PPI’ and ‘off PPI’, respectively, *P* = 0.177; *Figure 1C*). Similarly, the median time to diagnosis was 20.0 (14.3, 29.0) days in patients treated with PPI and 25.0 (17.8, 30.8) days in those without (*P* = 0.123; *Figure 1D*).

The primary initial symptoms in patients with and without PPI treatment included fever (60.0% vs. 58.6%, *P* = 1.00), chest pain or odynophagia (54.1% vs. 58.6%, *P* = 0.829), neurological symptoms (stroke or seizures) (44.7% vs. 37.9%, *P* = 0.665), and others (58.8% vs. 79.3%, *P* = 0.072) (*Figure 1E*).

### Complications and outcome

The complication rate did not differ between patients treated with PPI compared to those without (*Figure 1F*). Most frequently observed were severe sepsis or septic shock (53.2% vs. 73.1%, *P* = 0.108), coma (45.6% vs. 50.0%, *P* = 0.821), stroke or cerebral haemorrhage (19.0% vs. 38.5%, *P* = 0.062), gastrointestinal bleeding (16.5% vs. 19.2%, *P* = 0.768), cardiac tamponade (11.4% vs. 11.5%, *P* = 1.00), or others (15.3% vs. 13.8%, *P* = 0.222).

Mortality was high and comparable in both groups (65.9% for patients ‘on PPI’ vs. 65.5% for patients ‘off PPI’, *P* = 1.00; *Figure 1G*). A total of 7/85 (8.2%) vs. 4/29 (13.8%) and 8/85 (9.4%) vs. 2/29 (6.9%) patients experienced major or minor sequelae, respectively (*P* = 0.467 and *P* = 1.00). Only 13/85 (15.3%) vs. 4/29 (13.8%) patients had no sequelae (*P* = 1.00).

## Discussion

The key findings of the study are as follows:

1. The incidence of OF did not differ between centres with and without post-procedural PPI prescription.
2. Patients with and without PPI had comparable time to symptom onset, complication rate, and mortality.
3. The use of PPI following AF ablation has significantly increased over time.

Proton pump inhibitors are widely prescribed after left atrial ablation procedures to reduce gastric acidity, as gastroesophageal reflux is thought to contribute to the progression of ablation-induced oesophageal lesions and OF formation.^4,5,7,8^ Beyond potential prevention, PPI therapy might influence the time course and clinical presentation of OF. However, no difference in OF incidence was observed between centres with or without routine PPI use. Similarly, a study from Ugata *et al*.^9^ showed no reduction of mortality or severe oesophageal injury with prophylactic PPI. A recent substudy of the MADE-PVI trial suggested a protective effect in patients with pre-existing reflux oesophagitis.^10^ Nevertheless, no definite conclusions can be drawn from those observational or *post hoc* analyses, and no additional evidence supports or contradicts the use of PPI therapy.^8^ As the present study is a retrospective analysis exclusively in patients with OF, the independent and causal influence of post-procedural PPI prescription on the incidence of OF cannot be evaluated.

We observed comparable time to first symptoms, time to diagnosis, symptom burden, complications, and outcome, altogether questioning the effectiveness of PPI in OF prevention. Nevertheless, despite the lack of randomized data, empirical PPI treatment remains a reasonable approach due to its low cost and favourable safety profile, at least for thermal ablations. But trade-offs including relevant pharmacological interactions and an increased risk of infections should be considered.^6^

## Limitations

The retrospective nature of the study bears known limitations. Possible practice changes in routine PPI prescription during the observational period were not evaluated. The details on PPI therapy are not known and might have changed over the observational period.

## Data Availability

Data supporting the POTTER-AF study are curated at the Study Centre of the Department of Rhythmology, University Hospital Schleswig-Holstein, Germany. These data are not shared openly but are available on reasonable request from the corresponding authors.
